# Supplementary material for: Splice-Junction-Based Mapping of Alternative Isoforms in the Human Proteome
Source: Cell Rep. Author manuscript; Available in PMC 2020 Jan 15. (PMC6961840; doi:10.1016/j.celrep.2019.11.026)
Supplement: 2 [file NIHMS1546469-supplement-2.zip › DF1/PXD010154/Lung/Lung_12_CAMSAP3_ASPAAPADGAAPAQPSHAIAFCLK.pdf]

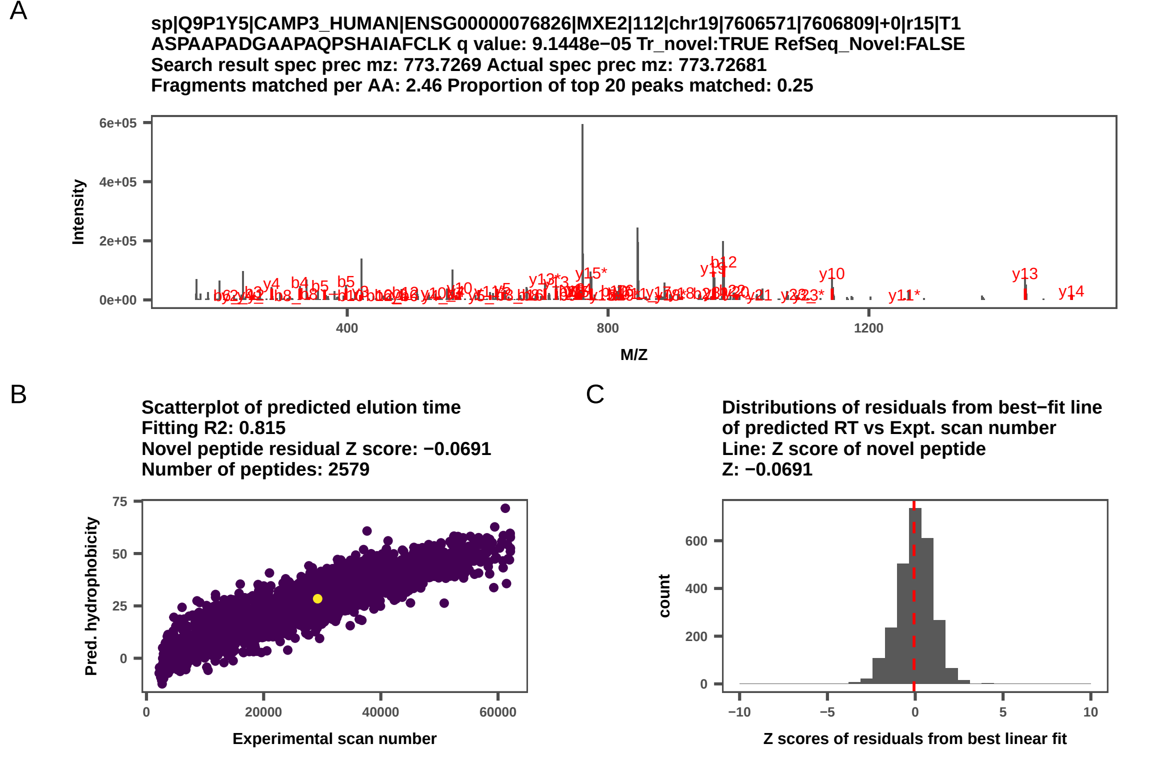
A
sp|Q9P1Y5|CAMP3_HUMAN|ENSG00000076826|MXE2|112|chr19|7606571|7606809|+0|r15|T1
ASPAAPADGAAPAQPSHAIAFCLK q value: 9.1448e−05 Tr_novel:TRUE RefSeq_Novel:FALSE
Search result spec prec mz: 773.7269 Actual spec prec mz: 773.72681
Fragments matched per AA: 2.46 Proportion of top 20 peaks matched: 0.25
Intensity
6e+05
4e+05
2e+05
0e+00
y4
b4
b5
y15*
b12
y10
y13
y14
y11*
400
800
1200
M/Z
B
Scatterplot of predicted elution time
Fitting R2: 0.815
Novel peptide residual Z score: −0.0691
Number of peptides: 2579
Pred. hydrophobicity
75
50
25
0
0
20000
40000
60000
Experimental scan number
C
Distributions of residuals from best−fit line
of predicted RT vs Expt. scan number
Line: Z score of novel peptide
Z: −0.0691
count
600
400
200
0
−10
−5
0
5
10
Z scores of residuals from best linear fit
